# Supplementary material for: Complexome profiling of the Chlamydomonas psb28 mutant reveals TEF5 as an early PSII assembly factor
Source: Plant Cell. 2025 Mar 17;37(6):koaf055. doi: 10.1093/plcell/koaf055 (PMC12164590; doi:10.1093/plcell/koaf055)
Supplement: koaf055_Supplementary_Data [file koaf055_supplementary_data.zip › psb28_interactive.zip!/plots/intensity/qualityPlots/peptides/NUOB14_Cre12.g555250.t1.2.html]

### Cre12.g555250.t1.2

Trivial name: NUOB14  
SeqCoverage [median]: 60.10  
Mass: 16.11 kDa  
  
 

| Sequence | MissedCleavage | Mass [Da] | wtIntensity | muIntensity | Score |
| --- | --- | --- | --- | --- | --- |
| AVEVAHQDKR | 1 | 1151.60 | 30634.10 | 28217.65 | 103.34 |
| FKEYKDVK | 2 | 1055.57 | 1419.26 | 2023.80 | 96.14 |
| GREEIETYLLMHK | 1 | 1617.82 | 15262.01 | 21688.15 | 79.63 |
| HHVVTEVVEPYYAK | 0 | 1669.85 | 15381.88 | 18687.29 | 175.48 |
| MEEMVSLK | 0 | 965.46 | 8154.06 | 7379.55 | 119.41 |
| VTTNSPFLDSFLSSGYAAIGVR | 0 | 2301.16 | 1308.79 | 0.00 | 65.45 |
| VVDLLIFK | 0 | 945.59 | 35962.46 | 23296.92 | 127.12 |
|  |  |  | 108122.6 | 101293.4 |  |
